# PARP enzymes and mono-ADP-ribosylation: advancing the connection from interferon-signalling to cancer biology

**DOI:** 10.1017/erm.2024.13

**Published:** 2024-08-27

**Authors:** Barbara Morone, Giovanna Grimaldi

**Affiliations:** Institute for Endocrinology and Experimental Oncology, National Research Council, Via Pietro Castellino 111, Napoli, Italy

**Keywords:** cancer-targeted therapy, interferon-signalling, mono-ADP-ribosylation, PARP inhibitors, PARPs

## Abstract

ADP-ribosyltransferases of the PARP family encompass a group of enzymes with variegated regulatory functions in cells, ranging from DNA damage repair to the control of cell-cycle progression and immune response. Over the years, this knowledge has led to the use of PARP1/2 inhibitors as mainstay pharmaceutical strategies for the treatment of ovarian, pancreatic, prostate and breast cancers, holding mutations in genes encoding for proteins involved in the DNA repair mechanisms (synthetic lethality). Meanwhile, the last decade has witnessed significant progress in comprehending cellular pathways regulated by mono-ADP-ribosylation, with a huge effort in the development of novel selective compounds to inhibit those PARPs endowed with mono-ADP-ribosylation activity. This review focuses on the progress achieved in the cancer field, delving into most recent findings regarding the role of a subset of enzymes – the interferon-stimulated PARPs – in cancer progression.

## Introduction

ADP-ribosylation is a reversible post-translational modification involved in several cellular processes, ranging from transcription, cell-cycle regulation and intracellular membrane transport to DNA-damage repair and stress-related responses (Ref. 1). The reaction consists in the transfer of an ADP-ribose moiety from the donor nicotinamide adenine dinucleotide (NAD^+^) to acceptor proteins and/or nucleic acids (Refs 2, 3); this reaction is catalysed by ADP-ribosyltransferases (ARTs), while being reversed by glycosylhydrolases. By removing ADP-ribose units from the modified targets, these enzymes ensure the dynamic and reversible nature of ADP-ribosylation, allowing cells to rapidly respond to different physiological and environmental stimuli (Ref. 4).

**Figure 1.**
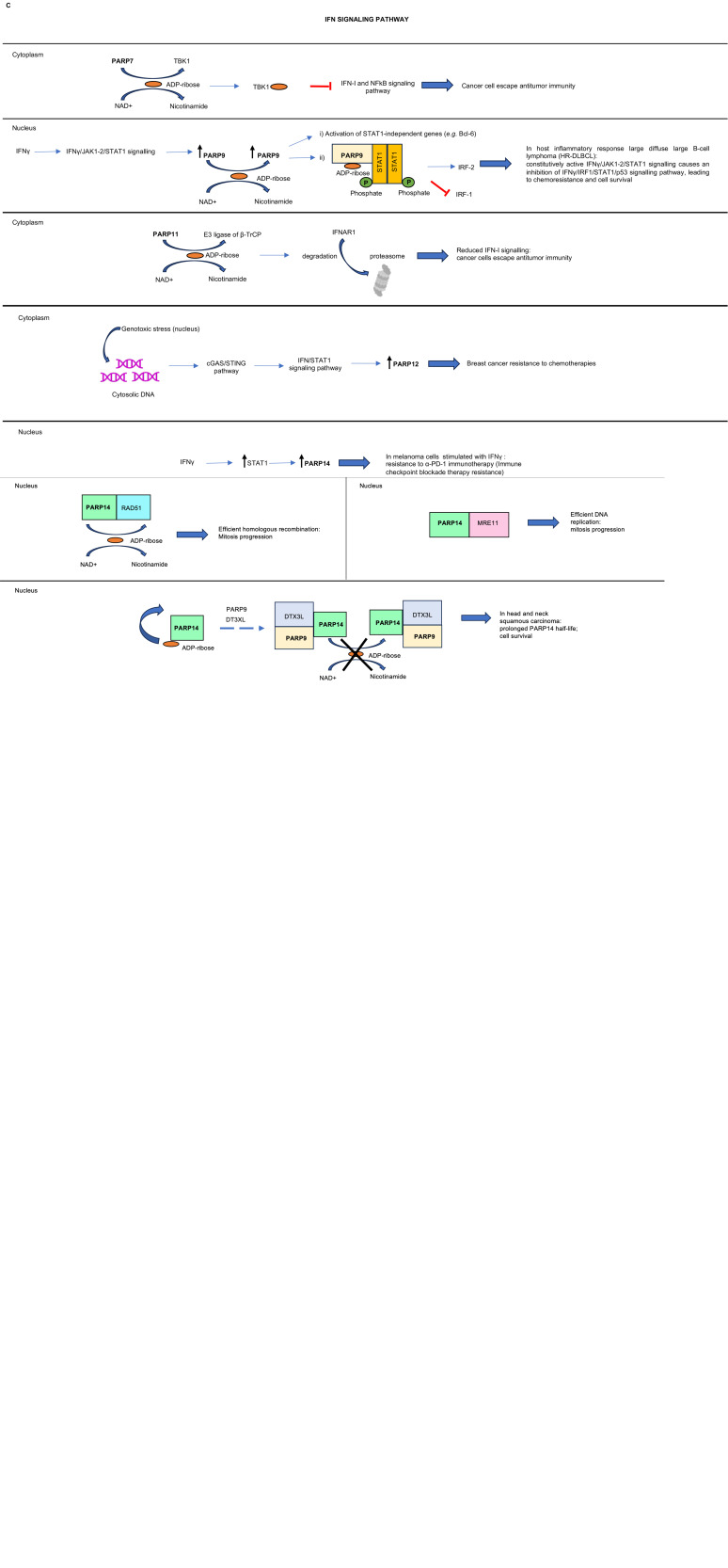
Schematic representation of key cellular processes (A, B, C) deregulated in cancer, in which PARP enzymes are involved. When indicated in the figure and specified herein, the mechanism is subcellular (nucleus or cytoplasm) or cellular specific. Question mark (?) indicates a molecular mechanism still unknown; arrows indicate upregulation (in black) or downregulation (in red). See text for details. (A) PARP7, PARP10 and PARP14 roles in cell-cycle regulation. From the top: *PARP7* upregulation in prostate cancer cells leads to an increased MARylation of its substrate, androgen receptor (AR), and a subsequent transcriptional hyperactivation of AR-target genes. *PARP7* MARylates and stabilises protein levels of FRA1, a component of the activator protein 1 (AP-I) transcription factor, involved in a variety of process including response to growth factors and cytokines. This event inhibits the transcription of IRF-1-target genes, such as MAVS and RIG-I, leading to an inhibition of IRF-3 activation and transcriptional repression of its target genes, responsible for apoptosis induction. *PARP10* is involved in the repair of DNA damage caused by replicational stress by interacting with PCNA (proliferating cell nuclear antigen). The high proliferative rate of cancer cells causes replicational stress with subsequent formation of stalled fork at DNA level, causing the block of replication. PARP10/PCNA interaction overcomes the replicational arrest. *PARP10* MARylates Aurora-A kinase during mitosis. This modification activates Aurora-A allowing transition from G2 to M phase and cell-cycle progression. *PARP10* MARylation of PLK1 inactivates the kinase, which in turn cannot phosphorylate PARP10. Reduced phosphorylated levels of PARP10 leads to activation of the NFk*β* pathway and enhanced cell proliferation. *PARP14* binds and stabilises the 3′ UTR of cyclin-D1 mRNA. This corresponds to an increase of cyclin-D1 protein levels, thus promoting cell-cycle transition from G1 to S phase. *PARP14* interacts with and MARylates the DNA repair protein RAD51, an event essential for an efficient homologous recombination repair therefore favouring cell survival. *PARP14* interacts with and activates MRE11, a nuclease involved in single-strand DNA degradation at stalled replicational forks, therefore overcoming DNA replication block. *PARP14* forms a complex with PARP9 and histone E3 ubiquitin ligase, DTX3L, in head and neck squamous carcinoma. This interaction blocks PARP14 auto-ADP ribosylation: the enzyme half-life is prolonged and promotes pro-survival signalling. (B) PARP7, PARP10, PARP12 and PARP14 are involved in cell growth and motility. From the top: *PARP7* MARylates *α*-tubulin in ovarian cancer cells; this modification stabilises microtubules, thus promoting cell motility and survival. *PARP10* MARylates and impairs Aurora-A phosphorylation, therefore avoiding endothelial-to-mesenchymal transition (EMT) progression. *PARP10* upregulation in head and neck tumours correlates with an increased phosphorylation of components of the PI3K/AKT and MAPK pathways (black arrows), activating a proliferative response (in figure, p-AKT, phosphorylated AKT serine/threonine kinase; p-SRC, phosphorylated proto-oncogene SRC, Rous sarcoma; p-38, phosphorylated P38 mitogen-activated protein kinase; p-RSK, phosphorylated ribosomal S6 kinase). *PARP12* interacts with FHL2, regulating its stability and causing the repression of TGF*β*1, a key inducer of EMT. *PARP14*-mediated MARylation of HDAC2 and HDAC3 is activated by interleukin-4 (IL-4), in colorectal cancer; this event causes inactivation of these deacetylases and therefore activation of the EP4 gene transcription, encoding for prostaglandin E2 receptor, which promotes proliferation and invasion of cancer cells. *PARP14* expression is increased by the serine/threonine kinase JNK2, in multiple myeloma. PARP14 interacts with JNK1, inhibiting its signalling, involved in proapoptotic process. (C) PARP7, PARP9, PARP11, PARP12 and PARP14 regulate IFN signalling. From the top: *PARP7* MARylation of TBK1 inhibits its kinase activity, leading to a repression of IFN-I and NFk*β* signalling pathways. *PARP9* overexpression in host inflammatory response large diffuse large B-cell lymphoma (HR-DLBC) depends on the continuous stimulation with interferon gamma (IFN*γ*), which causes a constitutively active IFN*γ*R-JAK1/2-STAT1 signalling pathway. Increased PARP9 levels can either directly enhance the expression of the STAT1-independent proto-oncogene BCL6 (i) or promote STAT1 phosphorylation on Y701 residue, therefore activating transcription of STAT1-dependent target oncogenes, such as IRF-2 (ii). The activated STAT1-complex also acts as a transcriptional repressor for the tumour suppressor gene IRF-1. Altogether these events cause an increase of HR-DLBCL cells proliferation, chemoresistance and survival. *PARP11* MARylates E3 ubiquitin ligase of *β*-TrCP; this modification causes ubiquitination and subsequent degradation of the IFN*α* receptor subunit IFNAR1, responsible for repression of the IFN-I signalling. *PARP12*: cytosolic DNA activates cGAS/STING pathway, leading to the activation of the expression of interferon-stimulated genes, such as PARP12. High PARP12 levels promote cell survival and breast cancer resistance to chemotherapy. *PARP14*: immunotherapy with *α*-PD-1, a monoclonal antibody which blocks the negative regulator of T-cell activation, PD-1, can cause an increase of IFN*γ* level and signalling in melanoma cells and overexpression of STAT1. STAT1 upregulates PARP14, causing immunotherapy resistance: this is reversed when PARP14 expression is impaired or its catalytic activity inhibited.

Intracellularly, ADP-ribosylation is mainly operated by enzymes of the PARP family, which consists of 17 members that, based on their catalytic activity, are grouped into mono-ARTs (PARP3, 4, 6–12, 14–16) or poly-ARTs (PARP1, PARP2 and PARP5a/5b); to date, only PARP13 is considered as an inactive enzyme (Ref. 1). PARPs can transfer either single or multiple units of ADP-ribose, and we will refer to mono-ADP-ribosylation (MARylation) or poly-ADP-ribosylation (PARylation), respectively. The last two decades have been characterised by improvements in the tools to detect the modification, leading to a wider understanding of the roles played by the different enzymes. More in details, mono-ARTs appear to be involved in the regulation of signalling pathways altered in cancers and therefore can be considered as innovative targets for integrated therapeutic approaches. This review provides a summary on the ‘state of the art’ regarding the role of mono-ARTs of the PARP family in cancer biology (Fig. 1), with a specific focus on members encoded by interferon-stimulated genes (ISGs; i.e. PARP7, 9–14) and their role in sustaining pro-survival signals and interplay with immune response.

## PARP7

PARP7 is a mono-ART involved in pathways that are often dysregulated in cancer cells and has a recognised role as a crucial modulator of innate immune signalling, acting as a repressor of interferon type I (IFN-I) pathway (Ref. 5). This function is achieved by MARylating and deactivating components of the pathway, such as the TANK-binding kinase 1 (TBK1) or likely other key effectors (Ref. 6). In cancer cells, where genomic instability leads to the accumulation of cytosolic nucleic acids, the presence of cytoplasmic DNA activates the cGAS-STING pathway and TBK1, and induces the expression and secretion of IFN-I. These secreted interferons at low concentration increase cancer cells proliferation, whereas at high concentration are detrimental for their survival, by enhancing the ISG expression and activation of the immune response. By negatively regulating the type I interferon response, PARP7 enables cancer cells to escape the immune surveillance. At the same time, loss of PARP7 function inhibits cancer cell expansion by regulating cell proliferation and death (Refs 5, 6, 7). Molecularly, as recently shown in breast and lung cancer cells, loss of PARP7 function causes reduced protein levels of the FRA1 transcription factor, leading to an upregulation of IRF1/3-dependent inflammatory and proapoptotic genes, responsible for the apoptosis induction (Ref. 7). Whether PARP7-mediated ADP-ribosylation of FRA1 also impact on other transcription machineries requires further investigations. Apparently, the role of PARP7 varies in different cancer types. In prostate cancer cells, PARP7 was identified as a positive regulator of the androgen receptor (AR) complex that contains AR itself, PARP9 and the ubiquitin E3 ligase DTX3L. PARP7-catalyzed AR MARylation favours complex formation and nuclear translocation, activating transcription of AR-regulated genes, required for prostate cell proliferation and development (Ref. 8).

An additional role of PARP7 emerged in ovarian cancer cells, where it MARylates *α*-tubulin of the microtubules, making the cancer cells prone to migration. Therefore, PARP7 loss or inhibition decreases cell growth and motility as demonstrated in different cell types (ovarian, cervical and kidney cancer cells) (Ref. 9).

Differently, studies performed in breast cancer cells indicated that PARP7 negatively regulates oestrogen receptor *α* signalling *in vitro* (Ref. 10), and additional research demonstrated that PARP7 knockdown promoted tumour growth in an MCF-7 xenograft model (Ref. 11). Notably, these studies were conducted without the presence of immune cells or a functional immune system, possibly explaining different results. The specific contribution of inhibiting PARP7 activity in tumour cells, immune cells or both has just started to be elucidated and requires wider analysis.

However, a recent study analysed the effect of PARP7 knockout in EO771 mouse mammary cancer cells and in a preclinical syngenic tumour model, utilizing wild-type and catalytically inactive mutant Parp7H532A mice, and showed that loss of PARP7 function modestly reduced cancer cell proliferation, while not affecting tumour growth in immunodeficient mice. Importantly, this outcome was fully reverted in immunocompetent mice, where PARP7 loss caused a robust reduction of tumour growth, which become further enhanced in mutant Parp7H532A recipient mice. These findings indicate that the absence of PARP7 enhances IFN-I and associated signalling pathways, resulting in heightened immune system targeting the tumours (Ref. 12).

The effectiveness of PARP7 inhibitor-induced tumour regression therefore relies on an intact IFN-I signalling pathway and an increased infiltration of CD8^+^ T cells (Refs 5, 6, 7, 8, 9, 10, 11, 12, 13). Currently, RBN-2397 stands as the sole PARP7 inhibitor undergoing clinical trials, both as a monotherapy for patients with advanced solid tumours (NCT04053673) and in combination with the immune checkpoint inhibitor pembrolizumab (NCT05127590) in patients with squamous cell carcinoma of the lung. Targeting PARP7 activity in immune cells emerges as a promising strategy to bolster the body's immune defences against tumours (Ref. 14).

## PARP9

PARP9, also known as B-aggressive lymphoma-1 (BAL1), was identified as a risk-related gene product in aggressive diffuse large B-cell lymphoma (DLBCL) and associated with interferon-gamma (IFN*γ*) gene expression and signalling. PARP9 represses the anti-proliferative and pro-apoptotic IFN*γ*-STAT1-IRF1-p53 axis, thus mediating proliferation, survival and chemo-resistance in DLBCL (Ref. 15). In addition, PARP9 has been found as part of a trimeric complex with DTX3L and PARP14, regulating proliferation, survival and chemo-resistance in metastatic prostate cancer cells, in a way dependent on STAT1 activation (Ref. 16). Similarly, PARP9/DTX3L also control the AR-mediated transcription in prostate cancer cells (Ref. 17). A similar role for PARP9 has been recently reported in head–neck squamous cellular carcinoma (HNSCC), where PARP9 forms a complex with PARP14 and DTX3L to promote survival signals (see below and (Ref. 18)). Therefore, a combined targeted inhibition of STAT1, PARP9, PARP14 and/or DTX3L could increase the efficacy of currently available treatment for prostate, DLBCL and other high-risk tumour types harbouring an increased STAT1 signalling.

## PARP10

PARP10 was the first mono-ART to be characterised and demonstrated to be a MYC-interacting protein and a negative regulator of NF*κ*B pathway in antiviral response (Ref. 19).

Later evidence proved that the enzyme is also involved in DNA damage repair and genomic instability, mostly through its interaction with PCNA, a master regulator of DNA replication and S-phase-coupled repair (Refs 20, 21, 22). In the nucleus, PARP10 cooperates with PCNA to induce DNA polymerization at stalled fork in condition of replicational stress. This role correlates with PARP10 overexpression in a large proportion of human tumours, where it promotes cellular transformation, potentially by alleviating cellular sensitivity to replication stress and favouring the restart of stalled replication forks (Refs 21, 22). The presence of a nuclear export sequence makes PARP10 capable to shuttle between nuclear and cytoplasmic compartments, therefore regulating both nuclear and cytoplasmic events. In the cytosol, for example, PARP10 guarantees a correct cell-cycle progression by regulating the G2 to M phase transition, through the MARylation and activation of the cell-cycle kinase Aurora A (Ref. 23). Another substrate of PARP10 in cancer cells is the cell-cycle kinase PLK1, which is overexpressed in a variety of cancers (*e.g*. liver, breast and lung cancers) promoting cell proliferation and metastasis. In particular, in hepatocellular carcinoma (HCC), PARP10-mediated MARylation of PLK1 significantly inhibits its kinase activity and related oncogenic functions. Indeed, PLK1 can phosphorylate PARP10, leading to an enhanced NF*κ*B transcriptional activity responsible for cancer progression (Ref. 24).

In other tumours, PARP10 deficiency correlates with an increase of cancer cell proliferation and migration: in this context, absence of PARP10 results in a reduced MARylation of the substrate Aurora A causing an increased cancer cell invasion and migration, without affecting cell-cycle progression. This indicates a regulatory activity of PARP10 on Aurora A that is dependent on the biological context (cell migration or cell-cycle progression) (Ref. 25).

Increased PARP10 expression has been recently shown to correlate with a poor prognosis in patients with oral squamous cell carcinoma, the most common type of head and neck cancer (Ref. 26). Here, PARP10 silencing hampered the PI3K and MAPK pathways. Although the role of PARP10 catalytic activity was not investigated, this study further confirms the importance of PARP10 as key regulator of cell proliferation and metastasis (Ref. 26).

Overall, although with different outcomes, dysregulation of PARP10 appears to be central in tumour progression, determining specific biological consequences strictly dictated by the cellular context and the substrates involved.

## PARP11

PARP11 is a mono-ART with roles in the regulation of immune response, specifically the IFN-I signalling. Differently from PARP7, that regulates IFN-I production, PARP11 controls the pathway by downregulating the levels of the interferon receptor chain 1, IFNAR1. IFNAR1 protein levels depend on its ubiquitination by *β*-TrCP-containing E3 ligase and followed by degradation (Ref. 27). *β*-TrCP expression is regulated by the Wnt signalling pathway and its activity is dependent on the MARylation mediated by PARP11. Therefore, increasing MARylation of the E3 ligase causes increased IFNR1 degradation, meaning a reduced IFN-I signalling. In Parp11 knock-out mice, IFNAR1 levels are kept at physiological levels, facilitating the activation of immune cells, in particular the cytotoxic T lymphocytes antitumoural activity (Ref. 28). Moreover, in colorectal cancer, PARP11 expression is decreased in a subset of T effector cells (CD103^+^ CD39^+^) (Ref. 29). Inhibition of PARP11 could therefore restore the signalling pathway mediated by IFNAR1 leading to the enhanced IFN-I and ISGs expression, making tumour cells more easily targeted by the immune system. On this line, the combination of PARP11 inhibition and the new therapy based on the generation of chimeric antigen receptor (CAR) T cells could improve the outcome of this approach: studies conducted on CAR T cells depleted of PARP11 show that these cells stably express the IFNAR1 on their surface and persist longer in blood and tumours in mice (Ref. 29).

## PARP12

PARP12 is a mono-ART mainly known as a component of stress granules, regulator of intracellular membrane transport and for its anti-viral effects (Refs 30, 31, 32). Along with these functions, PARP12 has been proposed to be involved in cancer biology, although its contribution is still controversial and elusive. PARP12 has been shown to suppress HCC metastasis, independent of its enzymatic activity. In this system, PARP12 interacts with the transcription factor FHL2, thereby regulating its stability and repressing transforming growth factor *β1* (TGF-*β*1) transcription, a potent driver of endothelial-to-mesenchymal transition (EMT) involved in cell migration and invasion of liver cancer cells (Ref. 33). In the breast cancer context, on the other hand, it has been observed that PARP12 is an active contributor of IFN/STAT1 signalling pathway involved in post-chemotherapy survival and re-growth of breast cancer cells (Ref. 34). Although the molecular mechanism underlying PARP12 role in breast cancer is not completely understood, it is considered a potential promising novel target in breast cancer therapy.

## PARP13

PARP13 is an RNA-binding PARP initially identified as an antiviral protein. Emerging from alternate splicing, PARP13 exists in four different isoforms (Refs 35, 36, 37); among them, the two best studied are PARP13.1 and PARP13.2. Although PARP13.2 does not code for a PARP domain, the PARP13.1 encodes a protein containing an inactive C-terminal PARP domain in addition to the CCCH zinc finger motif (Ref. 1). Most of the knowledge on this protein relates to its role as an anti-viral factor. Moreover, in immune cells, it regulates the response to TRAIL, a proapoptotic cytokine that once secreted can specifically target cancer cells to promote their death (Ref. 38). Indeed, several cancers (such as liver, bladder and colon cancers) show low levels of PARP13 expression compared with normal tissues suggesting that loss/decreased level of PARP13 favours the development and progression of different types of cancers.

Recently, a direct interaction between PARP1 and PARP13 has been shown, supporting the idea of a crosstalk among different PARPs, although the role of these interactions remains largely unknown. In the case of PARP1 and PARP13, it has been proposed that PARP1 is seised in the nucleus by the heat shock transcription factor 1 through the scaffold protein PARP13. In the presence of DNA damage, PARP1 is ADP-ribosylated and dissociates from this complex to relocate in specific genomic loci to promote repair of DNA lesions. Blocking this complex could affect the survival of the cancer cells, in particular in BRCA1-null mammary tumours (Ref. 39).

## PARP14

PARP14 was initially characterised as a positive regulator of STAT6 that increases the production of interleukin 4 (IL-4). In the absence of this cytokine, PARP14 forms a complex with histone deacetylases (HDAC2 and HDAC3), thus repressing the expression of target genes; upon IL-4 stimulation, PARP14 catalyses its own MARylation and that of HDAC2 and HDAC3, promoting gene transcription (Ref. 40). PARP14 role in tumour development and progression varies across cancer types, with implications in B-cell specific tumorigenesis, such as DLBCL and multiple myeloma. The role of IL-4 in cancer is controversial: in some studies, it acts as tumour suppressor whereas in others it promotes tumour progression. In colorectal cancer, MARylation of HDAC2 and HDAC3 mediated by PARP14 allows the expression of EP4, one of four prostaglandin receptors commonly upregulated in the tumour microenvironment exerting vital roles in stimulating cell proliferation, invasion and metastasis. Inhibition of PARP14 abrogates EP4 receptor activation, reducing proliferation, thus suggesting PARP14 as a potential target for colon cancer therapy (Ref. 41). PARP14 is also implicated in B-cell lymphoma where it inhibits JNK1 signalling, involved in cell apoptosis, and in acute myeloid leukaemia where it enhances the glycolysis and promotes cancer cell proliferation (Refs 20, 42). Along with its role in metabolism and immune signalling, PARP14 exerts important functions also in the maintenance of genome integrity, by promoting an efficient homologous recombination (HR) (Ref. 20). The study suggests that PARP14 MARylates RAD51, a key factor in HR, and this event is crucial for efficient HR; PARP14 depletion leads to persistent RAD51 foci, preventing a complete and successful HR (Ref. 43).

Moreover, a recent study analysed synthetic lethal interactions of PARP14, and identifies the ATR-CHK1 pathway as essential for the viability of PARP14-deficient cells, making them hypersensitive to inhibitors (Ref. 44). Similarly, PARP14 can modulate fork stability, specifically in BRCA1/2-deficient cells, a function mediated by PARP14 interaction with MRE11 in order to prevent excessive resection at stalled replication forks (Ref. 45).

Additionally, PARP14 is implicated in cell-cycle regulation, promoting G1/S phase transition *via* the retinoblastoma pathway. In particular, it was shown that PARP14 increases cyclin D1 levels, involved in G1/S transition both in normal and cancer cells, by binding to the 3′ UTR of cyclin D1 mRNA, stabilizing the transcript. When PARP14 is depleted, cells with functional Rb and p53/p21 pathway are blocked in G1 phase which causes cell-cycle arrest (Ref. 46).

More recently, PARP14 protein levels were found to be increased in HNSC and other tumour types, along with elevated protein levels of its interactors PARP9 and DTX3L. Depletion of PARP14, as well as depletion of PARP9 or DTX3L, reduced survival and proliferation of HeLa and HNSC cells. This effect was independent on PARP14 catalytic activity, although relies on a C-terminal domain required for the formation of the PARP14/PARP9/DTX3L heterotrimer. Mechanistically, this interaction inhibits PARP14 auto-ADP-ribosylation, favouring an increased PARP14 stability at protein level, necessary to mediate pro-survival signalling (Ref. 18).

Interestingly, and connected to the role of PARP14 in IFN-mediated signalling, tumours with an acquired resistance to immune checkpoint blockade therapy (such as immunotherapy targeting programmed cell death protein 1, referred as *α*-PD-1 therapy) show increased PARP14 mRNA levels, along with IFN*γ* genes. Treatment with the PARP14 inhibitor RBN012579, by enhancing the IFN*γ* signalling, re-sensitise these tumours to the PD-1 therapy, establishing PARP14 as an actionable target to reverse IFN*γ*-driven resistance mechanisms to immune checkpoint blockade therapy (Ref. 47).

Collectively, by promoting pro-survival pathways, inhibition of pro-apoptotic factor JNK1 and G1/S cell-cycle progression through cyclin D1 expression, PARP14 can be considered as a pro-survival factor, and a valuable promising drug target to be investigated. Currently, the PARP14 inhibitor RBN-3143 has entered phase I clinical trials as therapy for a range of inflammatory diseases, with an initial focus on atopic dermatitis (NCT05215808).

## Role of other PARPs in cancer biology

With this section we provide a snapshot of PARPs and MARylation in cancer cell development and progression, not dependent on the IFN-signalling.

**PARP3** is involved in DNA double-strand break repair and chromosomal rearrangements (Ref. 48). Overexpression of PARP3 in breast cancer patients correlates with an increase of tumour aggressiveness and a reduction of survival rate. Remarkably, PARP3 promotes TFG*β*-induced EMT and cell proliferation of triple-negative breast cancer by increasing the stability of mTORC2 complex (Refs 49, 50). Moreover, in glioblastoma cells, PARP3 acts to maintain the cytoskeletal microtubule stability and its absence markedly increases the sensitivity of glioblastoma cells to microtubule-destabilizing agents, providing a novel therapeutic strategy in brain cancer therapy (Ref. 51).

**PARP4** is one of the least studied PARPs and little is known about PARP4 functions and mechanism of action. The presence of the BRCA1 carboxy-terminal domain repeats, similar to those present in PARP1, suggests an involvement of this enzyme in DNA damage repair. In a study conducted on a cohort of patients affected by primary thyroid and breast cancers, PARP4 seems to act as a tumour suppressor gene (Ref. 52). More recently, PARP4 mRNA expression was found to be significantly upregulated in cisplatin-resistant ovarian cancer cell lines and depletion of its expression in cisplatin-resistant cell lines reduced cisplatin chemoresistance, restoring the cisplatin-induced DNA fragmentation (Ref. 53). PARP4 expression levels correlate with the DNA methylation status at its specific promoter site, a feature that could be exploited as a novel biomarker for predicting the response to cisplatin in ovarian cancer patients (Ref. 53).

**PARP6** has been shown to be involved in the genesis of different tumours; however, its mechanism of action is still controversial and not completely characterised. PARP6 was initially described as a negative regulator of cell proliferation. Indeed, according to both immunohistochemical and Kaplan–Meier analysis, PARP6-positive colorectal cancer correlated with a good prognosis, supporting a role of PARP6 as a tumour suppressor through its involvement in cell-cycle control and apoptosis induction (Refs 54, 55). These findings were further confirmed for HCC by subsequent studies, where PARP6 was found to negatively correlate with the degree of tumour differentiation of HCC. Mechanistically, PARP6 inhibited the Wnt/*β*-catenin signalling pathway, by promoting XRCC6 protein degradation (Ref. 56). On the same line, in breast cancer cells, pharmacological and genetic inhibition of PARP6 caused mitotic defects in breast cancer cells, leading to apoptosis induction (Ref. 57). Differently, PARP6 seems to have an opposite role in gastric cancer, where it promoted cell proliferation, migration and invasion, by activating the survivin pathway (Ref. 58). Collectively, these findings demonstrate a central role of PARP6 in cancer development and a novel drug target selective for a subset of tumours.

**PARP8** is localised at the nuclear envelope, except during mitosis where it localises at centrosomes and at spindle poles (Ref. 59). A specific function of PARP8 has not been described yet. Recently, in uveal melanoma, depletion of PARP8 correlates with a decrease in proliferation and migration rate of cancer cells (Ref. 60). Further studies are therefore required to identify pathways regulated by this enzyme.

**PARP16** is the only PARP that possesses a C-terminal trans-membrane domain associated with the nuclear envelope and the endoplasmic reticulum (ER). PARP16 plays a key role in the activation of ER stress sensors, PERK and IRE1*α*, involved in the unfolded protein response (Ref. 61). In *Drosophila*, it was discovered that MARylation mediated by PARP16 is also involved in the metabolic stress response (Ref. 62). PARP16 has been shown to MARylate ribosomal proteins, and its catalytic activity is supported by the NAD-synthetising enzyme NMNAT2. Inhibiting PARP16 enhances translation of specific mRNAs, causing ER-stress-induced cancer cell apoptosis (Ref. 63). The role exerted by PARP16 in the regulation of stress response and translation made it a target for new combinatorial therapies. Remarkably, in small cell lung cancer, treatment with the PARP1 inhibitor talazoparib, which also targets PARP16, is more efficient in reducing cancer cell survival. Moreover, PARP16 silencing reduces cell survival in combination with olaparib and adavosertib, a WEE1/PLK1 inhibitor. Collectively, these findings highlight a contribution of PARP16 in talazoparib-mediated mechanism of action and suggest PARP16 as a novel alternative pharmacological target in small cell lung cancer (Ref. 64).

## PARP inhibitors

Majority of the efforts in PARP inhibition in cancer therapies were initially focused on PARP1 because of its key role in DNA integrity maintenance. Olaparib was the first PARP inhibitor approved by the Food and Drugs Administration in 2014 and used in therapy to cure breast, ovarian and prostate cancers in patients carrying defects in HR machineries.

In this frame, the discovery of mono-ARTs as regulator of the IFN-I signalling in cancer progression is promoting continuous efforts to better characterise these enzymes and to generate new inhibitors to improve the outcome of the current therapies for the treatment of different cancers.

One of the challenges in the generation of selective inhibitors is due to the highly conserved catalytic domains shared among PARPs. In the last years, an important advance has been made for the synthesis of PARP7, PARP10 and PARP14 inhibitors, thus making them potential targets for new therapeutical approaches.

The PARP7 inhibitor RBN2397 is currently tested in phase-1 clinical trials, both in breast and lung cancer cells, enhancing the IFN-I signalling pathways negatively regulated by PARP7, and in prostate cancer cells, where PARP7 regulates the stability of AR, leading to an inhibition of prostate cancer cells growth in culture (Ref. 65). Moreover, it was also observed that the use of RBN-2397 inhibitor after PARP1 depletion enhanced IFN-I signalling, suggesting that the inhibition of PARP1 could enhance the antitumoural immunity of PARP7 (Ref. 66). This aspect has been recently reviewed by Cohen and coworkers and we refer the reader to this text for a complete overview on the activation of innate immunity mediated by PARP1 inhibitors (Ref. 14).

Exploring the intricate connections among PARPs, MARylation, cancer biology and IFN signalling is essential for advancing our understanding of these processes. Insights into these interactions may open avenues for the development of targeted therapies and interventions in cancer treatment.

## Abbreviations

AR, androgen receptor; ART, ADP-ribosyltransferase; ATR, ataxia telangiectasia and Rad3-related protein; BAL-1, B-aggressive lymphoma-1; BRCA1/2, breast cancer type 1 susceptibility protein 1/2; *β*-TrCP, beta-transducin repeat containing E3 ubiquitin protein ligase; CAR T, chimeric antigen receptor T cell; cGAS, cyclic GMP-AMP synthase; CHK1, checkpoint kinase 1; DLBCL, diffuse large B-cell lymphoma; DTX3L, deltex E3 ubiquitin ligase 3L; EMT, endothelial-to-mesenchymal transition; ER, endoplasmic reticulum; EP4, prostaglandin E receptor 4; FHL2, four and a half LIM domains 2; FRA1, FOS like 1, AP-1 transcription factor subunit; HCC, hepatocellular carcinoma; HDAC2/HDAC3, histone deacetylase 2/histone deacetylase 3; HNSCC, head–neck squamous cellular carcinoma; HR, homologous recombination; IL-4, interleukin 4; IFN-I, interferon type 1; IFN*γ*, interferon gamma; IFNAR1, interferon alpha and beta receptor subunit 1; IRE1*α*, inositol-requiring enzyme 1; IRF1/IRF3, interferon regulatory factor 1/interferon regulatory factor 3; ISG, interferon stimulated gene; JNK1, c-Jun N-terminal kinase 1; MAPK, mitogen-activated protein kinase; MARylation, mono-ADP-ribosylation; mTORC2, mTOR complex 2; NAD, nicotinamide adenine dinucleotide; NFk*β*, nuclear factor kappa B subunit 1; NMNAT2, nicotinamide mononucleotide adenylyl transferase 2; p53, tumour protein P53; PARylation, poly-ADP-ribosylation; PCNA, proliferating cell nuclear antigen; PD-1, programmed cell death protein 1; PERK, protein kinase R-like endoplasmic reticulum kinase; PI3K, phosphoinositide-3-kinase; PLK1, polo like kinase 1; RAD51, RAD51 recombinase; STAT1, signal transducer and activator of transcription 1; STAT6, signal transducer and activator of transcription 6; STING, stimulator of interferon response cGAMP interactor 1; TBK1, TANK-binding kinase 1; TGF-*β*1, transforming growth factor beta 1; TRAIL, TNF-related apoptosis-inducing ligand; XRCC6, X-ray repair cross complementing 6.
